# Examining bidirectional associations between sleep and behavior among children with attention‐deficit/hyperactivity disorder

**DOI:** 10.1002/jcv2.12157

**Published:** 2023-03-20

**Authors:** Craig A. Sidol, Stephen P. Becker, James L. Peugh, James D. Lynch, Heather A. Ciesielski, Allison K. Zoromski, Jeffery N. Epstein

**Affiliations:** ^1^ Division of Behavioral Medicine and Clinical Psychology Cincinnati Children's Hospital Medical Center Cincinnati Ohio USA; ^2^ Department of Psychology University of Cincinnati Cincinnati Ohio USA; ^3^ Department of Pediatrics University of Cincinnati College of Medicine Cincinnati Ohio USA

**Keywords:** ADHD, intraindividual variability, sleep efficiency, summer treatment program, total sleep time

## Abstract

**Background:**

Children with attention‐deficit/hyperactivity disorder (ADHD) have more sleep problems than their peers which contribute to behavioral and functional impairments. This study examines the bidirectional relationship between nightly sleep (i.e., total sleep time and sleep efficiency) and daily behavior of children with ADHD.

**Method:**

Forty‐three children (ages 6–13 [mean = 9.05, 54% male, 77% medicated]) participated in a 2‐week study during an ADHD Summer Treatment Program (STP). Sleep was measured with actigraphy. Behavior was assessed using STP clinical data and daily parent and counselor ratings of ADHD symptoms, oppositional defiant disorder behaviors, and emotion regulation (e.g., difficulty regulating emotional disposition and controlling emotions). We hypothesized that healthier night's sleep measured by actigraphy (i.e., sleep efficiency and total sleep time [TST]) would relate to less ADHD symptoms, less emotional dysregulation, and better academic performance the next day. Additionally, we hypothesized that less ADHD symptoms, less emotional dysregulation, and greater academic performance would relate to healthier sleep that night.

**Results:**

Higher nightly sleep efficiency was related to improved parent‐ratings of ADHD the next day (*R* ^2^ = 0.04, *p* = 0.04) and improved parent‐ratings of ADHD during the day lead to higher sleep efficiency that night (*R* ^2^ = 0.002, *p* = 0.02). Higher rates of daily assignment completion were related to higher sleep efficiency at night (*R* ^2^ = 0.035, *p* = 0.03). TST was not related to any behavioral outcomes.

**Conclusion:**

Sleep efficiency may be more relevant than TST to behavioral performance the next day. Additionally, a bidirectional relationship exists between sleep efficiency and parent ratings of ADHD. Findings highlight the importance of assessing for manifestations of poor sleep efficiency, waking minutes, and wakings after sleep onset when diagnosing and treating ADHD.

**key Points**

- Children with attention‐deficit/hyperactivity disorder (ADHD) have more sleep problems than their peers which contribute to behavioral and functional impairments.
- Sleep efficiency may be more relevant to behavioral performance than total sleep time.
- A daily bidirectional relationship exists between sleep efficiency and parent ratings of ADHD.

## INTRODUCTION

Approximately 70% of children with attention‐deficit/hyperactivity disorder (ADHD) experience sleep problems (Sung et al., 2008). Documented ADHD‐related sleep problems include shorter sleep duration, longer sleep onset latency, more night wakings, lower sleep efficiency, greater daytime sleepiness, and more intraindividual variability of sleep/wake patterns, when compared to typically developing children (Becker et al., 2017; Cortese et al., 2009).

In pediatric ADHD samples, sleep has been associated with ADHD symptoms, emotion regulation, and academic performance. Regarding ADHD symptoms, parent‐reported sleep problems assessed with daily sleep diaries are strongly linked to inattentive and hyperactive‐impulsive symptom severity (Owens et al., 2009). Also, both greater frequency of total arousals during sleep and greater frequency of arousals from slow wave sleep assessed with polysomnography are related to greater severity of parent‐rated hyperactivity/immaturity and restless/disorganized behaviors (Stephens et al., 2013).

Poor sleep quality is also related to poor emotion regulation in children with ADHD. Namely, children with ADHD who have more parent‐rated difficulties initiating and maintaining sleep have more parent‐rated emotional problems (Mulraney et al., 2016) and more frequent and severe tantrums (Hiscock et al., 2007). In addition, parent‐reported difficulty falling asleep and restless sleep is related to higher parent‐ratings of irritability, anger, and temper tantrums in children with ADHD (Waxmonsky et al., 2017).

Sleep problems also appear to exacerbate academic difficulties in children with ADHD (Sciberras et al., 2015). Child‐rated frequency of night wakings was associated with lower math achievement scores among adolescents with ADHD (Cusick et al., 2018). Also, greater adolescent‐report of daytime sleepiness predicted higher parent‐ and teacher‐rated academic difficulties in youth with ADHD (Langberg et al., 2013).

The aforementioned studies have established associations between sleep problems and behavioral and functional impairments in children with ADHD using global measurements of sleep problems and behavioral outcomes. Sleep has generally been assessed with sleep questionnaires, sleep diaries, or actigraphy data characterizing the child's sleep over the previous week or months. Likewise, ADHD behavior, emotion regulation, and academic outcomes have primarily been assessed using weekly or monthly ratings across these various domains. Hence, much of what we know about the relation between sleep and its impact on child behavior/functioning in children with ADHD reflects a general association between summary sleep and summary behavior. Such associations ignore the daily variability in sleep and behavior that is common among children with ADHD (Becker et al., 2017). Moreover, it does not allow for more causal inferences. Namely, the directionality of the described association between sleep and behavior is unknown. Poor night's sleep may lead to poor next‐day behavior. Alternatively, poor behavior may lead to a poor night's sleep. For example, children with ADHD may have a poor night's sleep due to behavioral difficulties prior to bedtime (Owens et al., 2000). Some recent sleep intervention trials have demonstrated that improving sleep can improve ADHD symptoms and some associated outcomes (e.g., social behavior and emotion regulation) suggesting a causal link between sleep and ADHD behavior (Hiscock et al., 2015; Keshavarzi et al., 2014), though studies with night‐to‐day analyses remain lacking.

Bidirectional relationships may also exist. Studies examining day‐to‐day associations which may inform directionality have, to date, only been conducted in neurotypical samples, with mixed results. Van Dyk et al. (2016) found no associations between sleep duration and externalizing behavior, though a bidirectional relation was found between actigraphy‐assessed sleep duration and daily mood ratings. Kouros and El‐Sheikh (2015) found sleep was not associated with mood the following day, though a more negative mood during the day predicted higher levels of activity during sleep and a later sleep onset that night. Another study focused on academics found adolescents who stayed up late, thus having less sleep, had more academic problems the following day (Gillen‐O’Neel et al., 2013).

Though research among neurotypical children is limited and presents mixed findings for the relationship between sleep and outcomes, a stronger relationship may be present among children with clinical diagnoses (i.e., ADHD) as children with ADHD have higher levels and more variability in nightly sleep problems and behavioral outcomes than neurotypical children (Becker et al., 2017). Furthermore, it has been posited that children with clinical diagnoses (i.e., ADHD) may be more vulnerable to the effects of poor sleep than neurotypical peers due to having a lower levels of emotion regulation and impulse control (Van Dyk et al., 2016). This study is among the first to examine the bidirectional relationship of objectively measured sleep (actigraphy) and behavior in children with ADHD across a range of behavioral domains. We hypothesized that healthier night's sleep measured by actigraphy (i.e., sleep efficiency and total sleep time [TST]) would relate to less ADHD symptoms, less emotional dysregulation (e.g., difficulty regulating emotional disposition and controlling emotions), and better academic performance the next day. Additionally, we hypothesized that less ADHD symptoms, less emotional dysregulation, and greater academic performance would relate to healthier that sleep night.

## METHOD

### Participants

This study was conducted in the context of an ADHD Summer Treatment Program (STP; Pelham et al., 1996). Fifty‐six children ages 6–13 years participated over the course of two summers (2018 and 2019). This age range corresponds to the age of participants enrolled in the STP and corresponds to the age range when ADHD symptoms tend to be most prevalent (Döpfner et al., 2015). An ADHD diagnosis and assessment of other current comorbidities was confirmed through administration of the Kiddie Schedule for Affective Disorders and Schizophrenia for School‐Age Children‐Present and Lifetime Version (Kaufman et al., 1996), administered to the primary caregiver by trained examiners.

### Setting

The STP is a summer day camp for children with ADHD. Children attended the program for 8 h each day (8:30 AM–4:30 PM), 5 days a week, for 7 weeks. The STP is a setting in which children engage in both academic and recreational activities in groups of 10–12 same‐age peers. Groups participated daily in classroom periods, recreational sports, swimming, and social activities. A primary counselor is assigned to each child and is responsible for providing individual feedback on daily progress toward the child's goals. For the duration of the program, STP staff members implemented a manualized behavioral management system which includes the detailed recording of behavioral data throughout the treatment day (Pelham et al., 1996).

### Procedures

This study was approved by the Cincinnati Children's Hospital Medical Center Institutional Review Board. Parents provided written informed consent and children provided assent. Children participated in the study for a 2‐week period, beginning and ending on a Friday. Week one of the STP was excluded from the research study as the first week of the program is often considered a “honeymoon” week where children and staff adjust to the behavioral intervention.

During the 2‐week period, children wore an actigraph and both parents and STP staff completed daily ratings (see below for details). STP staff completed the same rating forms three times a day (10:30 AM, 12:30 PM, and 4:30 PM), Monday–Thursday. Parents completed their ratings daily around 9:00 PM. Parents also completed a daily sleep diary (see below). STP behavioral data was only collected Monday–Thursday as Fridays are not considered a typical treatment day.

### Measures

#### Daily measures

##### Sleep diary

Parents completed an adaptation of the consensus sleep diary (Carney et al., 2012). The diary included bedtime, sleep latency, rise time, and daily medication use. Diaries were used to corroborate actigraphy data.

##### Actigraphy

The Micro‐Motionlogger SleepWatch^®^ (Ambulatory Monitoring, Inc., Ardsley, NY) collects movement data that are entered into an algorithm to infer sleep and wake states (Sadeh, 2011). TST was calculated as the total minutes the child is asleep while in bed. Sleep efficiency was calculated by dividing TST by time in bed (i.e., initial onset to final offset).

##### Inattention/Overactivity with Aggression (IOWA) Conners Rating Scale (Waschbusch et al., 1998)

Both parents and STP primary counselors completed the 10‐item Inattention/Overactivity with Aggression Conners Rating Scale. Questions on the four‐point scale measure the severity of inattention, impulsivity, and oppositionality that a child is exhibiting (0 = not at all, 3 = very much). The rating scale yields two 5‐item subscales that measure ADHD and oppositional defiant disorder (ODD) symptoms, with total scores calculated by summing the five items for each subscale.

##### Emotion regulation checklist (ERC; Shields & Cicchetti, 1997)

An abbreviated, 15‐item version of the emotion regulation checklist (ERC) with only emotion regulation items was used in the current study and completed by both parents and STP primary counselors. Items on this four‐point subscale measure a child's daily emotional disposition and their ability to regulate their emotions (0 = never, 3 = almost always) and were summed to calculate a total emotion regulation score. Higher ratings denoted greater emotion dysregulation.

##### STP Outcomes

STP clinical data provides data on 24 operationalized behaviors. To simplify the analyses, total points earned, classroom rule violations, average assignment completion, and average assignment accuracy were used. Children earned points in the program for following activity rules, refraining from exhibiting disruptive behaviors, demonstrating compliance, and initiating pro‐social behaviors. Rule violations in the classroom occurred when a child violated one of the classroom rules. During a 30 min seatwork period, children are given three assignments to complete (math, reading, and a journal). Children receive points for completion only if the entire assignment is finished. Assignment accuracy is calculated by the number of correct responses divided by the total number of questions. Assignment difficulty and number of items were modified over time depending on child skill level.

### Missing data

All children included in the analyses had at least five nights of actigraph data (total missing nights: *M* = 1.28, SD = 1.76, range 0–7; missing nights related to STP and counselor ratings: *M* = 0.42, SD = 0.76, range 0–3), consistent with literature examining the reliability of actigraphy data (Short et al., 2017). Thirteen children were excluded from the final analyses as a result of not having 5 days of actigraph data (technological issues: *n* = 8; non‐compliance: *n* = 5), resulting in a final sample size of 43 children. Excluded children did not differ from included children in terms of baseline sociodemographics. Imputation was conducted via Blimp (Enders et al., 2018) to account for the remaining missing daily data due to child forgetting to wear the watch (9% of nights) incomplete/lost parent ratings (16%) or missing STP data (7.5%; child was not present at the STP).

### Analyses

Data from each summer were merged into a single data set.

Two‐level (Level 1: subject; Level 2: day) multi‐level modeling structure was used for all analyses. Primary analyses examined the association between nightly sleep actigraphy (e.g., TST and sleep efficiency) and daily behavioral and academic functioning during the STP. Using linear mixed models, day was included as a repeated measures variable with 8 days (i.e., 2 weeks including only Monday–Thursday) for STP outcomes and counselor ratings and 14 days for parent ratings. Further, similar models were conducted to include the daily behavioral outcome and day as an independent variable and sleep efficiency as the dependent variable to examine whether daily behavior might affect that night's sleep. The current study examined the significance of each model in both directions. An overall *R* ^2^ was computed for all models with significant effects.

## RESULTS

Sample demographics, ADHD presentation, and medication status are presented in Table 1. All results for bidirectional relationships between sleep and behavior are presented in Table 2.

**TABLE 1:** Participant demographics.

	*n* = 43
Age: Mean (SD) (range)	9.05 (1.78) (6–13)
Sex: Male *n* (%)	30 (54%)
KSADS diagnosis: *n* (%)	
ADHD inattentive presentation	21 (49%)
ADHD hyperactive/impulsive presentation	2 (5%)
ADHD combined presentation	22 (46%)
ODD	11 (26%)
GAD	2 (5%)
MDD	1 (2%)
OCD	1 (2%)
Medication status	
No medication	10 (23%)
Stimulant only	15 (35%)
Stimulant + non stimulant	5 (12%)
Stimulant + mood	5 (12%)
Stimulant + non stimulant + mood	4 (9%)
Non stimulant only	1 (2%)
Non stimulant + mood	2 (5%)
Mood only	1 (2%)
Melatonin sleep aide	10 (23%)

Abbreviations: ADHD, attention‐deficit hyperactivity disorder; GAD, generalized anxiety disorder; MDD, major depressive disorder; Mood, medications that generally treat mood disorders; OCD, obsessive compulsive disorder; ODD, oppositional defiant disorder.

**TABLE 2:** Regression results examining the bidirectional relationship between sleep efficiency and daily behavior.

Dependent variable	Sleep efficiency								Sleep minutes							
	Night to day				Day to night				Night to day				Day to night			
	*b*	S.E.	*t*	*p*	*b*	S.E.	*t*	*p*	*b*	S.E.	*t*	*p*	*b*	S.E.	*t*	*p*
STP behavior																
Points earned	4.25	4.19	1.01	0.31	0.00	0.00	−0.25	0.81	1.29	28.17	0.05	0.96	0.00	0.00	0.80	0.42
Seatwork completion	0.20	0.12	1.64	0.10	0.06	0.03	2.13	0.03	0.48	0.81	0.60	0.55	0.00	0.01	0.80	0.42
Seatwork accuracy	0.13	0.16	0.77	0.44	0.03	0.02	1.40	0.16	−0.02	1.11	−0.18	0.86	0.03	0.02	1.40	0.16
Seatwork rule violations	−0.01	0.03	−0.37	0.71	0.02	0.09	0.22	0.83	0.03	0.23	0.13	0.9	0.01	0.02	0.41	0.68
Parent ratings																
IOWA Conners ADHD	−0.06	0.03	−2.03	0.04	−0.27	0.12	−2.38	0.02	−0.10	0.14	−0.66	0.51	−0.02	0.02	−1.31	0.20
IOWA Conners ODD	0.00	0.03	−0.07	0.94	−0.11	0.09	−1.19	0.24	0.07	0.16	0.45	0.66	−0.02	0.01	−1.51	0.13
ER checklist	−0.12	0.07	−1.80	0.07	−0.08	0.04	−1.74	0.08	−0.33	0.35	−0.94	0.35	−0.01	0.01	−1.72	0.09
Staff ratings																
IOWA Conners ADHD	−0.04	0.03	−1.36	0.18	0.19	0.14	1.31	0.19	−0.14	0.11	−1.29	0.20	−0.03	0.13	−0.21	0.84
IOWA Conners ODD	0.01	0.03	0.27	0.79	0.18	0.13	1.30	0.19	−0.01	0.12	−0.10	0.92	0.02	0.07	0.30	0.77
ER checklist	0.06	0.07	0.92	0.36	0.01	0.06	0.24	0.81	0.01	0.28	0.03	0.98	0.01	0.03	0.29	0.77

Abbreviations: ADHD, attention‐deficit hyperactivity disorder; ER, emotion regulation; IOWA, Inattention/Overactivity with Aggression; STP, summer treatment program.

### Sleep efficiency and behavior

A bidirectional relationship was observed between nightly sleep efficiency and daily parent ratings of ADHD, such that lower nightly sleep efficiency led to higher parent ratings of ADHD the next day (*R* ^2^ = 0.04, *p* = 0.04) and that higher parent ratings of ADHD symptoms during the day led to lower sleep efficiency that night (*R* ^2^ = 0.002, *p* = 0.02). Moreover, nightly sleep efficiency was not associated with daily points earned, rule violations, or seatwork accuracy. No relation was observed between nightly sleep efficiency and seatwork completion the next day. A positive relationship was found between daily seatwork completion and sleep efficiency (*R* ^2^ = 0.035, *p* = 0.03), such that greater seatwork completion led to higher sleep efficiency that night. Nightly sleep efficiency was not associated with daily points earned, rule violations, or seatwork accuracy. No relationship in either direction was observed between sleep efficiency and ratings of ODD, or emotion regulation, regardless of rater. Nor were any relationships in either direction observed between sleep efficiency and counselor ratings of ADHD.

### TST and behavior

No relationship in either directions was observed between nightly TST and ratings of ADHD, ODD, or emotion regulation, regardless of rater. Additionally, no bidirectional relationship was observed between nightly TST and any STP behavioral or academic outcome.

## DISCUSSION

The current study expanded upon prior research which has demonstrated an association between average (i.e., past week, past month) parent ratings of sleep and ADHD symptoms in children, by examining the potential bidirectional relationship between nightly sleep (i.e., sleep efficiency and TST) and daily behavior. In support of our hypotheses, a bidirectional relationship was found between sleep efficiency and parent ratings of ADHD, and daily assignment completion was related to improved sleep efficiency at night. Contrary to our hypotheses, no bidirectional relationship was found for TST and any behavioral outcome. Additionally, no bidirectional relationship was found for sleep efficiency and counselor ratings, parent ratings of emotion regulation, parent ratings of oppositional behavior, assignment accuracy, points earned, or rule violations.

These findings suggest that sleep efficiency may be more relevant to behavior in children with ADHD than TST. This may be due to how these sleep variables are calculated. TST is the total minutes the child is asleep while in bed and sleep efficiency is total minutes the child is asleep (i.e., TST) divided by time in bed (i.e., initial onset to final offset). Unlike sleep efficiency, the TST metric does not contain information about waking minutes. Waking minutes may indicate several sleep‐related factors (e.g., restless sleep, more nighttime arousals) that are related to both poor sleep as well as night‐to‐night variability in sleep among children with ADHD (Cortese et al., 2009; Cusick et al., 2018; Stephens et al., 2013). Indeed, polysomnographic research has documented that higher numbers of waking minutes are associated with more ADHD symptoms (Stephens et al., 2013). Although not reported in this manuscript, we conducted analyses using waking minutes assessed using actigraphy and found a similar pattern of findings as were reported for sleep efficiency. Thus, sleep efficiency may be a more consistent predictor of behavior than TST as sleep efficiency is a metric that accounts for several components of disrupted sleep (i.e., nighttime awakenings, restless sleep) that tend to be problematic in children with ADHD.

As this is the first study to our knowledge to explore the bidirectional relationship between nightly sleep and daily behavior in an ADHD sample, it is interesting to compare our results with similar prior studies conducted with neurotypical children. Our findings are supported by Van Dyk et al. (2016) suggestion that individuals with clinical diagnoses (i.e., ADHD) may be more vulnerable to the impact of poor nightly sleep than typically developing individuals. It is possible this relationship was observed by parents due to their observing morning and evening behaviors that occur outside the context of the STP and when medication and STP effects may also not be present.

Moreover, low nightly sleep efficiency is likely the result of greater frequency of sleep arousals, likely from slow wave sleep, which are associated with higher parent‐ratings of ADHD (Stephens et al., 2013). The relationship between lower daily parent‐rated ADHD symptoms followed by higher sleep efficiency that night may be explained by children being less resistant around their bedtime (Owens et al., 2000) experiencing higher sleep efficiency that night. It is also possible that children with fewer ADHD symptoms may also have better sleep hygiene and routines before bedtime, which facilitate better sleep quality.

Although the relation between sleep and academic performance has been documented among children with ADHD (Cusick et al., 2018; Langberg et al., 2013), most studies interpret this association as indicating poor sleep impacts academic performance and fail to consider the alternative. However, some literature on neurotypical children might offer insights into why higher academic performance may lead to higher sleep efficiency at night. One potential explanation is the relationship between poor sleep and children's self‐ratings of feelings of academic competence and global success (Becker, 2014). It is possible that children with greater seatwork completion experience increased self‐efficacy related to schoolwork which could lead to higher sleep efficiency that night. Additionally, greater perceived schoolwork/homework difficulty has been associated with insufficient sleep that night (Pagel et al., 2007). Furthermore, difficult academic tasks have been shown to tax the prefrontal cortex and have been associated with worse sleep (Curcio et al., 2006). Therefore, it is possible that children with greater seatwork completion experienced a less taxing day academically which facilitated higher sleep efficiency that night.

Despite an established link between poor sleep and emotion dysregulation (Palmer & Alfano, 2017) research examining the bidirectional relationship between sleep and next‐day mood in neurotypical children is limited and yields mixed results. Two studies which found associations between nightly sleep and next day's emotional functioning (e.g., anxiety and negative affect; Fuligni & Hardway, 2006; Van Dyk et al., 2016) relied on child‐report. When parent‐report of emotional functioning is used as an outcome, associations between nightly sleep and emotional functional were not found (Kouros & El‐Sheikh, 2015; Van Dyk et al., 2016). The present study also relied solely on parent report and similarly did not find associations between sleep and emotional functioning. Some research suggests that parents of children with ADHD may not recognize internalizing symptoms in their child because of the salience of their child's externalizing problems or parental psychological problems (Chi & Hinshaw, 2002; Webster‐Stratton & Herbert, 1994). Thus, it is possible that nightly variability of sleep is more strongly associated with self‐reported emotional behavior or multi‐rater‐report (De Los Reyes et al., 2015) and is not associated with parent‐reported emotional behavior alone.

Several limitations deserve consideration in interpreting the study results. First, we posit that the STP intervention as well as medication may partially explain our null findings. The STP provides a unique opportunity to collect behavioral ratings across the day, but it is also an active treatment program. Due to its use of constant, highly‐manualized behavioral contingencies and salient rewards when children are at the STP, there are low rates of problematic ADHD‐, ODD‐, and emotion regulation‐related behaviors (Fabiano et al., 2014). In fact, studies with adults have found that the detrimental effects of restricted sleep are reduced when behavioral incentives are introduced (Hsieh et al., 2010). It is possible that the STP environment attenuated behavioral variability which may have reduced our ability to detect a relationship between nightly sleep and daily STP behavior.

Another possible explanation for the apparent absence of an association between nightly sleep and daily STP behavior in our study was the use of medication by some of our study participants. ADHD medications clearly impact behavior as well as sleep. In addition to attenuating the effects of sleep deprivation, stimulant use reduces both ADHD symptoms (Corkum et al., 2020) and emotional lability (Childress et al., 2014) among children with ADHD. Much of the prior research examining sleep and ADHD required children to be off medication during the course of the study (Owens et al., 2009; Stephens et al., 2013). In contrast, the current study was comprised of only 23% medication naïve participants and did not require a wash‐out period nor children to be off‐medication. Thus, it is possible that, similar to the argument presented above regarding the STP environment, medication effects on behavior and emotion regulation may have attenuated our ability to detect any effects that nightly sleep may have had on daily STP behavior. Unfortunately, we were not able to control for medication in our methods and analyses as medication status during the 2‐week period the child participated in the current study may have changed. Furthermore, stimulant medication adherence decreases during the summer when school is not in session (Brinkman et al., 2018). In our sample 15.17% of daily medication data was not complete and our imputation models were unable to converge with medication status in the model. As such, future studies may benefit from exploring the bidirectional relationship between sleep and behavior absent of intervention (i.e., outside the context of the STP, using a non‐medicated sample).

Future studies may also benefit from conducting the research while school is in session. Although the STP hours are similar to a school day, summer schedules differ from the school year (i.e., homework and associated academic pressures, extracurricular activities). Thus conducting a similar study during the school year to determine possible bidirectional effects between sleep and behavior in youth with ADHD is necessary.

Our study sample was potentially limited in terms of size, diversity, and symptom severity. These limitations were a result of the STP context. Socioeconomic diversity of the sample was potentially limited as a result of the STP's high out‐of‐pocket cost. Moreover, children with comorbid conduct disorder and aggression were excluded by the STP director. It is possible that the association between sleep and emotion regulation and oppositional behavior is more readily identified in children with more severe behaviors. We were unable to explore the impact of additional predictor variables (e.g., ADHD symptom severity, sex, race, and ethnicity) as imputation models would not converge with covariates in the model. We encourage future studies, with potentially larger samples, to directly assess and include covariates in future work. Furthermore, while some of our effects were statistically significant, the effect sizes were rather small. Reasons for these small effects may be due to reliability of daily assessment of children's behavior or the fact that the study was conducted within the highly effective STP intervention which may have restricted the range of observable behavior. In addition, some studies have found that subjectively assessed sleep is more strongly associated with behavior than objectively assessed sleep (e.g., Lemola et al., 2011). This may be due to correspondences or biases introduced by having the same person rate sleep and behavior. It will be important for future studies to incorporate multi‐method assessment of sleep, particularly as rating scale measures of sleep are most feasible for use in most clinical practice settings.

Current diagnostic procedures for ADHD do not include an assessment of sleep problems; however, up to 70% of individuals with ADHD experience problems with sleep (Sung et al., 2008) and restless sleep was previously a symptom for diagnosing ADHD in the Diagnostic and Statistical Manual of Mental Disorders, 3rd edition (APA, 1980). Results of this study suggest that lower average sleep efficiency is related to daily behavior in children with ADHD and that lower nightly sleep efficiency is related to greater parent‐rated symptoms of ADHD the next day. Thus, clinicians would benefit from ruling in/out a comorbid sleep disorder when assessing for ADHD as comorbid sleep problems may impact ADHD symptom presentation and severity. The importance of assessing for sleep is bolstered by recent evidence from an experimental sleep restriction study that found shortened sleep duration to be a causal contributor to increased ADHD symptoms (Becker et al., 2019). We did not screen for organic sleep disorders (e.g., obstructive sleep apnea, periodic limb movement disorder) in our sample.

Finally, clinicians who treat children with ADHD would benefit from integrating a sleep treatment component into intervention. Indeed, low intensity behavioral interventions such as sleep hygiene practices have been associated with fewer sleep problems and a reduction of ADHD symptoms among children with ADHD (Hiscock et al., 2015; Peppers et al., 2016). More intense behavioral interventions have also been associated with fewer sleep problems among children with ADHD (Corkum et al., 2011) and these interventions may improve sleep as well as ADHD and associated co‐occurring psychopathology symptoms and impairments (Becker et al., 2022).

## AUTHOR CONTRIBUTIONS

**Craig A. Sidol**: Conceptualization; Data curation; Formal analysis; Methodology; Project administration; Writing – original draft. **Stephen P. Becker**: Conceptualization; Methodology; Supervision; Writing – original draft. **James L. Peugh**: Data curation; Formal analysis; Resources; Software; Supervision. **James D. Lynch**: Data curation; Writing – review & editing. **Heather A. Ciesielski**: Data curation; Resources; Writing – review & editing. **Allison K. Zoromski**: Data curation; Resources; Writing – review & editing. **Jeffery N. Epstein**: Conceptualization; Methodology; Resources; Supervision; Writing – original draft.

## CONFLICT OF INTEREST STATEMENT

Stephen P. Becker is a joint editor for JCPP Advances. The remaining authors have declared that they have no competing or potential conflicts of interest.

## ETHICAL CONSIDERATIONS

This study was approved by the Cincinnati Children's Hospital Medical Center Institutional Review Board. Parents provided written informed consent and children provided assent.

## Data Availability

Data are available from the corresponding author upon reasonable request.
